# Comment on “Effects of Scheduled Exercise on Cancer-Related Fatigue in Women with Early Breast Cancer”

**DOI:** 10.1155/2015/264695

**Published:** 2015-03-30

**Authors:** Isla Veal, Nicola Peat, Gareth Jones

**Affiliations:** Physiotherapy Department, Guy's and St Thomas' Foundation Trust, London SE1 9RT, UK

It is with interest that we read the recently published article titled “Effects of Scheduled Exercise on Cancer-Related Fatigue in Women with Early Breast Cancer” [1].

We are interested in the reported levels of physical activity levels found with the IPAQ-SF in this cancer population. Research has shown that physical activity levels in the cancer population at diagnosis are in line with noncancer population, followed by deterioration as they proceed through cancer treatment [2].

Therefore we were surprised when it was reported in this article that 100% of the control and intervention group met exercise recommendations before chemotherapy, immediately after chemotherapy, and 18 weeks later using the IPAQ-SF tool.

The IPAQ has been reported in the past to allow overreporting of physical activity, resulting in inflated reports meeting exercise recommendations. A recent systematic review [3] reported the IPAQ to be responsible for degrees of overreporting of physical activity levels of between 36% and 173% above the objective measure. Compared to other self-reporting tools in the cancer population, in which the 7-day Physical Activity Recall demonstrated overreporting by 13%, the IPAQ-SF overreported by 247% [4]. Multiple reasons for overreporting in the IPAQ-SF have been cited in research, for instance, difficulty in recall of average time of physical activity [5], multiple domain reporting including nonleisure activities [6], and lack of participant comprehension of terminology [5].

Our recent research has also demonstrated the possibility of inflated IPAQ-SF results with large proportion of the cancer population meeting exercise recommendations at the end of treatment in excess of the normal population.

In addition to all the mentioned reasons for overreporting particularly in the cancer population, another possible cause is the inclusion of walking in the calculation for meeting exercise recommendations. Walking as a form of exercise has been reported to be a very common choice of exercise in the cancer population [2, 7] with 81–88% of a sample making walking their chosen activity [8]. As intensity of walking is not specified in the IPAQ-SF questionnaire, low intensity walking (<3 Metabolic Equivalents) may be erroneously reported and included in meeting exercise recommendation calculations which only include intensities of “moderate” and “vigorous” [9]. Also, through self reporting “walking” activities separate to “moderate” intensity activities there is a chance for duplication of physical activity reporting. There are warnings to not duplicate, however, these are not always heeded. Overreporting may also be further exacerbated in the cancer population in the health care setting with the desire to give socially acceptable responses.

The IPAQ-SF has demonstrated overreporting of physical activity levels in the past and therefore is at risk of incorrectly identifying an active population. The cancer population may be at greater risk of misrepresentation by the IPAQ-SF due to the popularity and ease of walking as a form of physical activity throughout cancer treatment. From the work we have recently completed and from the evidence base we have reservations in using this tool to accurately identify the inactive population within cancer survivors.

What are the thoughts on the overreporting of physical activity with the use of the IPAQ tool? Could this have any wider impact on the conclusions drawn from this research?
